# High curvature promotes fusion of lipid membranes: Predictions from continuum elastic theory

**DOI:** 10.1016/j.bpj.2023.04.018

**Published:** 2023-04-18

**Authors:** Gonen Golani, Ulrich S. Schwarz

**Affiliations:** 1 Institute for Theoretical Physics and BioQuant Center for Quantitative Biology, Heidelberg University, Heidelberg, Germany

## Abstract

The fusion of lipid membranes progresses through a series of hemifusion intermediates with two significant energy barriers related to the formation of stalk and fusion pore, respectively. These energy barriers determine the speed and success rate of many critical biological processes, including the fusion of highly curved membranes, for example synaptic vesicles and enveloped viruses. Here we use continuum elastic theory of lipid monolayers to determine the relationship between membrane shape and energy barriers to fusion. We find that the stalk formation energy decreases with curvature by up to 31 k_B_T in a 20-nm-radius vesicle compared with planar membranes and by up to 8 k_B_T in the fusion of highly curved, long, tubular membranes. In contrast, the fusion pore formation energy barrier shows a more complicated behavior. Immediately after stalk expansion to the hemifusion diaphragm, the fusion pore formation energy barrier is low (15–25 k_B_T) due to lipid stretching in the distal monolayers and increased tension in highly curved vesicles. Therefore, the opening of the fusion pore is faster. However, these stresses relax over time due to lipid flip-flop from the proximal monolayer, resulting in a larger hemifusion diaphragm and a higher fusion pore formation energy barrier, up to 35 k_B_T. Therefore, if the fusion pore fails to open before significant lipid flip-flop takes place, the reaction proceeds to an extended hemifusion diaphragm state, which is a dead-end configuration in the fusion process and can be used to prevent viral infections. In contrast, in the fusion of long tubular compartments, the surface tension does not accumulate due to the formation of the diaphragm, and the energy barrier for pore expansion increases with curvature by up to 11 k_B_T. This suggests that inhibition of polymorphic virus infection could particularly target this feature of the second barrier.

## Significance

Lipid membranes continuously undergo fusion and fission events during the life of cells, e.g., at the plasma membrane, in endosomes, and at synapses. They also play a central role in the life cycle of enveloped viruses. Despite experimental observations and molecular dynamics computer simulations that show the importance of membrane curvature for fusion, a systematic understanding is lacking. Here we use continuum elastic theory to numerically calculate the fusion reaction between compartments of different geometries, sizes, and curvatures to determine the two main energy barriers to fusion, namely stalk and fusion pore formation. We apply our results to several biologically important systems ranging from synaptic vesicles to enveloped viruses.

## Introduction

**Figure 1:**
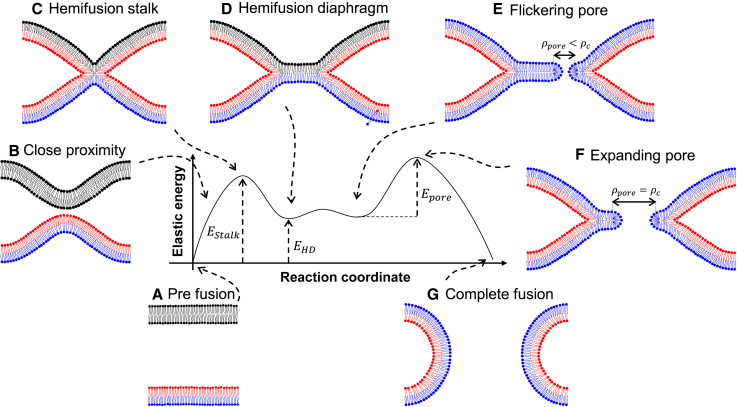
A sketch of the canonical fusion pathway. The reaction progress is shown clockwise from (*A*–*G*). When forced into proximity by fusion proteins, such as the SNARE-proteins, the two membranes form the hemifusion stalk, and the lipids in their outer (proximal) leaflets start to mix (*red color*). Crossing the barrier of hemifusion stalk formation is the first major barrier for membrane fusion. It then expands to the hemifusion diaphragm. Pore formation now also allows the lipids of the inner (distal) leaflets to mix (*blue color*), but fusion only succeeds if the second major barrier is crossed, namely pore expansion beyond a critical radius. To see this figure in color, go online.

Lipid membrane fusion is a crucial step in various biological processes such as fertilization, muscle cell formation, intercellular and extracellular trafficking, and infection with enveloped viruses. These diverse biological fusion events share a common pathway involving a universal series of lipid rearrangement steps (reviewed in (1,2,3,4,5,6,7)). The fusion process starts with designated fusion proteins, like the SNARE-family (8), bringing a small patch of the two fusing compartments to a 1–2 nm distance (Fig. 1 *A* and *B*). At this proximity, the membranes experience strong repulsive forces originating mainly from hydration forces (9), electrostatic repulsion (10), and membrane undulations (11). If these repulsive forces are overcome by the forced proximity and additional perturbations in the lipid membranes, the two opposing proximal monolayers merge to form a hemifusion connection called the hemifusion stalk (Fig. 1 *C*). It is generally accepted that the stalk constitutes a metastable minimum in the free energy landscape and that a significant energy barrier in the fusion reaction, *E*_*stalk*_, with a typical magnitude of several dozens of k_B_T has to be overcome to reach it (12,13,14,15). Computer simulations indicate that apart from lipid deformations, also hydration forces play an important role in this barrier (16). At this point in the reaction, the lipids in the outer (proximal) monolayers mix, which can be used experimentally to identify this state. Once formed, the hemifusion stalk radially expands to an equilibrium radius by bringing the two distal monolayers into contact along a joint mid-plane, an intermediate known as the hemifusion diaphragm (14,17) (Fig. 1 *D*). The diaphragm is mechanically stressed due to the strong tilt and splay deformations, with the maximal stress near the diaphragm rim. As a result, transient membrane pores tend to form there (18,19,20) (Fig. 1 *E*). The expansion of these pores is promoted by the membrane stress, *σ*, and resisted by the cost of forming and expanding the toroidal pore rim, *λ* (21,22,23,24). The ratio between the pore rim line tension and the membrane stress results in a critical radius *ρ*_*c*_∼*λ*/*σ*. Smaller pores tend to close, whereas larger ones tend to expand. The energy needed to create a pore with the critical radius *ρ*_*c*_ is the pore formation energy barrier, *E*_*pore*_*∼λ*^2^/*σ* (Fig. 1 *F*). Therefore, once a membrane pore forms, it flickers in size below the critical radius due to thermal energy until it eventually crosses the energy barrier and completes the fusion reaction (Fig. 1 *G*) (1,25,26). Computer simulations indicate that alternative pathways might exist for the formation of the fusion pore: for example, instead of undergoing radial expansion, the stalk could elongate and the pore could form in its vicinity (14,27,28,29,30). Here, however, we restrict our discussion to the canonical fusion pathway, as depicted in Fig. 1.

The time needed to complete the reaction depends on the magnitude of the energy barriers and the forces applied by the fusion proteins. It varies between hundreds of milliseconds in SNARE-mediated fusion (31) to seconds or minutes in cell-cell (32,33) and viral (34,35,36) fusion. Because of the importance of fusion in various biological processes, the magnitude of these energy barriers was estimated in numerous studies, primarily focusing on lipid composition (19,37,38), membrane mechanical properties (39,40), and tension (41,42). In addition, the geometry of the fusing membrane is gaining attention as a modulator of the fusion reaction pathway. For example, it was recently found that enveloped viruses with tubular shapes are more infectious than spherical ones, suggesting that the membrane fusion between the tubular virus envelope and the planar cell membrane might be faster (43). SARS-CoV-2 has been described to fuse mainly with the microvilli of epithelial cells of the upper airways (44). Furthermore, tubular membrane morphology was also found to be relevant in fertilization since sperm cells preferably fuse with the female egg cell at the highly curved microvilli structures and not on the flatter membrane surface (45). Lastly, vesicle size was found to influence synaptic vesicle fusion, with a linear dependence between the vesicle radius to the logarithm of the fusion pore formation time (46). Therefore, collective experimental evidence indicates that the fusing compartment geometry influences the energy barriers in the fusion pathway, but a theoretical framework to explain these experimental observations in a universal manner is still missing.

Previous studies relating membrane shapes and fusion rate focused on the role of membrane curvature in initiating fusion and stalk formation. It was proposed that membrane curvature promotes stalk formation since the stress related to the bending of the fusing membrane’s proximal monolayers is reduced by forming the stalk (47,48,49,50). This prediction was verified by experiments showing faster lipid mixing in small liposomes than in larger ones (51). Similar results were also obtained using computer simulations (16,18,52,53), which found a lower stalk energy in vesicle-vesicle or vesicle-to-flat membrane fusion compared with fusion between two flat membranes. In particular, coarse-grained MD simulations found that the hydration repulsion opposing stalk formation is reduced for curved membranes (16). Although curvature is now well established to promote stalk formation, its role in the later stages of diaphragm expansion and fusion pore formation is much less well understood. These stages are particularly interesting in the context of membrane shapes since their evolution is governed by internal membrane stress and not by the direct action of the fusion proteins, which must act from the outside of the diaphragm (54). Therefore, the stress related to the membrane’s curvature is expected to influence the transition to the hemifusion diaphragm and the formation of the fusion pore. Experimental evidence of that was found in liposome-based fusion assays that showed a substantial increase in the pore formation rate in small vesicles compared with large vesicles (51,55). However, more recent electron microscopy data showed that the fusion of small vesicles results in a stable extended hemifusion diaphragm of 5–10 nm radii (36,56,57).

In order to address both significant barriers in the canonical fusion pathway of curved membranes on the same footing and to investigate the role of curvature for the whole process, here we use continuum elastic theory to predict how stalk and pore formation energies vary as a function of the shapes of the fusing compartments. We show that the two intermediates have very different physics, but high curvature promotes fusion in both cases. We also spell out how viruses might have evolved to take advantage of these characteristics, particularly in the case of polymorphic viruses. Our results also suggest ways to inhibit viral membrane fusion. Finally, we discuss some of the limitations of the continuum elastic theory.

## Materials and methods

#### Lipid monolayer elasticity

**Figure 2:**
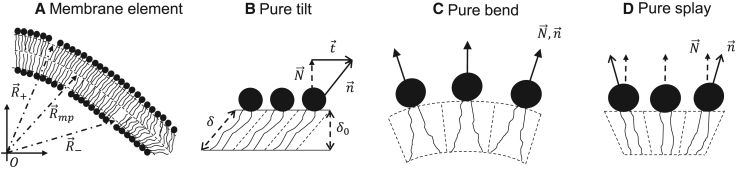
Lipids and membranes geometry. The lipid monolayers are described using tilt-splay theory. The membrane comprises two monolayers joint along the mid-plane. Definitions are as follows: $\vec{N}$, normal to monolayer plane; $\vec{n}\text{,}$ lipid director pointing from lipid tails to the headgroup; $\vec{t,}$ tilt vector (Eq. 2); *δ*_*0*_, the undeformed distance from the membrane mid-plan to the monolayer dividing plane; δ, the distance due to tilt deformation. (*A*) A membrane element with both tilt and splay deformations. ${\vec{R}}_{mp}$ is a vector pointing from the origin to the membrane mid-plane, and ${\vec{R}}_{-}$ and ${\vec{R}}_{+}$ are vectors to lower and upper monolayer dividing planes, respectively. (*B*) A lipid monolayer with pure tilt, constant tilt, and no splay deformations. (*C*) A lipid monolayer with pure bend and no tilt, $\vec{N}=\vec{n}$. (*D*) A lipid monolayer with pure splay and no geometrical bending, $\vec{\nabla }\cdot \vec{N}=0$, where the splay originates from the tilt change.

We model the lipid membrane using the well-established theory of lipid splay, tilt, and saddle splay (58,59,60,61). The membrane is composed of two monolayers that connect at a mid-plane, ${\vec{R}}_{mp}$ (Fig. 2 *A*). The orientation of the lipids in each monolayer is given by the local lipid director vector ${\hat{n}}_{\pm }$. The subscript ± represents the upper and lower monolayers, respectively. The monolayer dividing plane is located at

(Equation 1) $${\vec{R}}_{\pm }={\vec{R}}_{mp}+\delta \cdot {\hat{n}}_{\pm }\text{.}$$

The local lipid’s tail length, from membrane mid-plane to monolayer dividing plane, is given by *δ* and referred to hereafter as “tail length.” The lipid deformations of tilt, splay, and saddle splay are derived from ${\vec{R}}_{\pm }$ and ${\hat{n}}_{\pm }$.

Lipid tilt quantifies the magnitude of shear in the parallel direction to the plane of the monolayer, defined as follows (59,60):

(Equation 2) $$\vec{t}=\frac{\vec{n}}{\vec{n}\cdot \vec{N}}-\vec{N,}$$

with $\vec{N}$ being the normal to the monolayer dividing plane, ${\vec{R}}_{\pm }$. The lipid tilt can be related to the length of the lipid tails by

(Equation 3) $$\delta =\delta_{0}\sqrt{1+{\vec{t}}^{2}.}$$

*δ*_0_ is the length in the undeformed configuration. Lipid splay and saddle splay are generalizations of total and Gaussian curvatures in the presence of lipid tilt, respectively. They are derived from the lipid splay tensor,

(Equation 4) $${{\tilde{b}}_{\alpha }^{\beta }=\nabla }_{\alpha }n^{\beta }\text{,}$$

where the subscripts and superscripts denote the covariant and contravariant components in the local coordinate basis of the monolayer dividing plane, ${\vec{R}}_{\pm }$ (60). Lipid splay is the trace of the splay tensor,

(Equation 5) $$\tilde{J}={\tilde{b}}_{\alpha }^{\alpha }\text{,}$$

and lipid saddle splay is its determinant,

(Equation 6) $$\tilde{K}=\det\;{\tilde{b}}_{\alpha }^{\beta }\text{.}$$

In the absence of tilt, the splay is the sum of the monolayer’s two principal curvatures, $J=c_{1}+c_{2}$, and the saddle splay is their product, $K=c_{1}\cdot c_{2}$, where *c*_*1*_ and *c*_*2*_ are the principal curvatures of the dividing plane. The energy density up to quadratic order in tilt and splay is given by (58,60)

(Equation 7) $$u_{m}=\frac{1}{2}\kappa_{m}{\tilde{J}}^{2}-\kappa_{m}\tilde{J}J_{sm}+{\bar{\kappa }}_{m}\tilde{K}+\frac{1}{2}\kappa_{t}{\vec{t}}^{2}+\gamma_{m}\text{.}$$

The reference state is a flat tilt-less membrane. The bending rigidity of the monolayer, *κ*_*m*_, has a typical value of 5–15 k_B_T (62). The mean lipid intrinsic curvature, *J*_*sm*_, is the averaged intrinsic curvature of its constituting lipids (63,64,65),

(Equation 8) $$J_{sm}=\sum_{i=1}^{N}\zeta_{i}\varphi_{i}\text{.}$$

Here, N is the total number of lipid components. $\zeta_{i}$ and $\varphi_{i}$ are the intrinsic curvature and the mole fraction of the *i*’th lipid component, respectively. *J*_*sm*_ value ranges from −0.1 nm^−1^ in pure DOPC membrane (66,67), typically used in artificial membranes, to −0.28 nm^−1^ in cholesterol-rich membranes, such as the cell plasma membrane (68). The saddle-splay modulus, ${\bar{\kappa }}_{m}$, and tilt modulus, $\kappa_{t}$, cannot be directly measured and are estimated based on theoretical considerations and computer simulations. The ratio between saddle-splay modulus and bending rigidity, $\chi ={\bar{\kappa }}_{m}/\kappa_{m}$, is in the range of −1 to 0 (69,70), and the ratio between the bending rigidity to tilt modulus gives a tilt decay length of $l=\sqrt{\kappa_{m}/\kappa_{t}}$, typically between 1 and 1.5 nm (71,72). Unless indicated otherwise, we use *l* = 1.2 nm and *χ* = −0.5. *γ*_m_ is the monolayer tension that originates from the stretching of the lipid in the lateral direction (61):

(Equation 9) $$\gamma_{m}=K_{m}\frac{A_{m}-A_{m0}}{A_{m0}}\text{.}$$

*K*_m_ is the monolayer stretching-compression modulus with typical values of 80–160 mN/m (73), *A*_m_ is the monolayer area, and *A*_m0_ is the relaxed area of the monolayer. The membrane tension is the sum of its monolayer’s tensions ($\gamma_{\pm }$ for upper and lower monolayer, respectively),

(Equation 10) $$\gamma =\gamma_{+}+\gamma_{-}\text{.}$$

The overall elastic energy is given by the integration of the monolayer energy density (Eq. 7) over the area of the monolayers dividing planes,

(Equation 11) $$U=\int u_{+}dA_{+}+\int u_{-}dA_{-}\text{.}$$

Here, *u*_±_ and *dA*_±_ are energy densities (Eq. 7) and monolayer dividing-plane area elements of the upper and lower monolayers.

#### Description of the hemifusion intermediates

**Figure 3:**
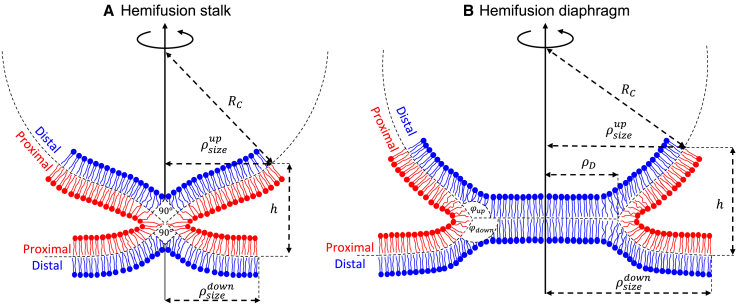
Description of hemifusion states between a flat membrane and a spherical vesicle with a radius $R_{C}$. The upper and lower fusion site sizes, $\rho_{size}^{up}$ and $\rho_{size}^{down}$, are defined as the radial distance from the center of the diaphragm to where the tilt deformation vanishes and the geometrical bending at the fusion site’s edge matches the surrounding compartment. The distance between the edges of the upper and lower fusion sites is *h*. (*A*) Hemifusion stalk. The angle between the mid-plane at the stalk center is fixed at 90°. (*B*) Hemifusion diaphragm. The diaphragm radius, $\rho_{D}$, is the distance from the diaphragm center to the three-way junction. The angles between the mid-planes at the diaphragm rim are given by *φ*_*up*_ and *φ*_*down*_. They are not fixed and are subject to energy minimization. *h*, $\rho_{size}^{up}$, and $\rho_{size}^{down}$ can assume different values at the stalk and hemifusion diaphragm and are also subject to energy minimization. The blue lipids are the distal monolayers, and the red lipids are the proximal monolayers. To see this figure in color, go online.

We consider fusion between flat, spherical, and cylindrical compartments. We explicitly calculate the combinations of spherical-spherical, flat-flat, spherical-flat, and cylindrical-flat fusion events. The fusion site has an axially symmetric configuration in spherical-spherical, flat-flat, and flat-spherical configurations. The symmetry is a quarter turn in configurations involving cylindrical membranes. The fusion site size is defined as the distance from the fusion site center at which the tilt deformations vanish. There the membrane has only pure bending deformation that matches that of the surrounding compartment (Fig. 3). Since there is no general up-down symmetry, this distance is different in the two compartments, given by $\rho_{size}^{up}$ and $\rho_{size}^{down}$, respectively (Fig. 3). In spherical-spherical and flat-flat, where up-down symmetry exists, we set $\rho_{size}={\rho_{size}^{up}}_{=}$ $\rho_{size}^{down}$. When no axial symmetry exists, the fusion site size has a quarter-turn angular symmetry. The distance between the fusion compartments is given by *h*. Unless indicated otherwise, we assume that fusion protein detaches and freely diffuses after the fusion initiation and does not exert forces on the membrane. Therefore, $\rho_{size}^{up}$, $\rho_{size}^{down}$, and *h* can assume their optimal value freely.

#### The hemifusion stalk elastic energy

We calculate the elastic energy of the hemifusion stalk. The hemifusion stalk is formed by the merger of the two proximal monolayers. The two fusing membranes form a 90° angle in the center of the stalk to prevent voids in the hydrocarbon tail region (13) (Fig. 3 *A*). As a result, the lipids are strongly sheared and splayed in the vicinity of the stalk. The stalk energy is the minimum energy needed to create the stalk from the prefusion state. We find it by numerically minimizing the sum of elastic energies (Eq. 11) in the upper and lower compartments, $U_{Elastic}^{up}$ and $U_{Elastic}^{down}$, while not allowing the diaphragm to expand (*ρ*_D_ = 0):

(Equation 12) $$E_{stalk}=\min\;{\left[U_{Elastic}^{up}+U_{Elastic}^{down}\right]}_{\rho_{D}=0}-U_{0}^{up}-U_{0}^{down}\text{.}$$

$U_{0}^{up}$ and $U_{0}^{down}$ are the prefusion elastic energies of the upper and lower compartment given by

(Equation 13) $$U_{0}^{spherical}=16\pi \kappa_{m}\left[1-\delta_{0}J_{sm}\right]+8\pi {\bar{\kappa }}_{m}$$

(Equation 14) $$U_{0}^{cylindrical}=2\pi \kappa_{m}\frac{R_{c}L}{R_{c}^{2}-\delta^{2}}$$

for the spherical (Eq. 13) and cylindrical (Eq. 14) compartments, respectively. The energies are given with respect to noninteracting compartments. The curvature radius of the compartment, defined as the curvature radius of the membrane mid-plane, is $R_{c}$ (Fig. 3 *A* and *B*). The proximal and distal mean lipid intrinsic curvature is $J_{sm}$. The length of the cylindrical compartment is *L,* and we omitted the elastic energy of the tubular membrane spherical ends because we assume that the fusion only involves the cylindrical part. Since the energy in Eq. 7 is with respect to a flat monolayer, a flat compartment has zero energy, $U_{0}^{flat}=0$. We explicitly assume that there is no preexisting lateral stretching in the monolayers. We further assume that the proximal monolayers can exchange lipids due to fast lateral flow during stalk formation, but the distal monolayers cannot since the existence time of the stalk is much shorter than the typical time of lipid flip-flop. In other words, when calculating the tension in Eq. 9, we consider the proximal monolayer a continuous one but the two distal monolayers separately. Finally, unless indicated otherwise, we assume that the volume inside the compartments is fixed.

#### The hemifusion diaphragm

The hemifusion diaphragm is spontaneously created by bringing the two distal monolayers into contact along the mid-plane of the double membrane (Fig. 3 *B*). The driving force for this process is the relaxation of the splay and tilt deformations. The rim of the diaphragm is the three-way junction between the newly formed diaphragm and the two surrounding fusing membranes. The rim has a circular shape with radius *ρ*_*D*_ in axially symmetric configurations, but in general, we allow the diaphragm rim to assume an elliptical shape in configurations with no axial symmetry. The junction is characterized by the two angles between the diaphragm and the two compartments, *φ*_*up*_ and *φ*_*down*_, respectively (Fig. 3 *B*), which can also have an angular dependency. We allow the diaphragm to bend in configurations with no up-down symmetry (spherical-flat and cylindrical-flat). The energy of forming the hemifusion diaphragm is calculated similarly to the hemifusion stalk, except that no constraint is imposed on the radius *ρ*_*D*_,

(Equation 15) $$E_{HD}=\min\left[U_{Elastic}^{up}+U_{Elastic}^{down}\right]-U_{0}^{up}-U_{0}^{down}\text{.}$$

Since the monolayers are continuous in the diaphragm rim, we require in our computation that the lipid director and tilt are the same on both sides of the junction. In addition, the expansion of the hemifusion diaphragm results in lateral stretching-compression of lipids, which can be partially relaxed due to lipid flip-flop over time. Therefore, we also calculate the diaphragm shape after a long time when lateral stretching stresses are relaxed.

#### Membrane-membrane interaction

We calculate the elastic energy of the hemifusion intermediates under the assumption that they are not subjected to an external force as provided, e.g., by SNARE-proteins. Therefore, the initial distance between the fusion compartment is sufficiently large to neglect short-ranged hydration and van der Waals forces, which cannot be included easily in continuum elastic theory anyway. Membrane undulation still contributes at these 5–10 nm distances, but it is in the order of a few k_B_T (11), and we consider it small compared with the elastic contributions. The short-range forces near the highly bent stalk or hemifusion diaphragm rim are accounted for in the bending modulus, κ_m_ (40). Neglecting the direct effect of the short-ranged forces is justified as long as the main energy contributions result from the deformations of the lipids.

#### Fusion pore formation

The fusion pore is formed by the merger of the two distal monolayers in the diaphragm and the subsequent expansion of the resulting membrane pore (74). The driving force for this process is the removal of the lipids from the highly stressed regions in the diaphragm and their migration to the less stressed surrounding membranes. The stress difference is given by

(Equation 16) $$\sigma =u_{+}\left(\rho ,\varphi \right)+u_{-}\left(\rho ,\varphi \right)-2u_{0}\text{.}$$

With $u_{dia}^{+}$ and $u_{dia}^{-}$ the stress in the upper and lower diaphragm monolayers and *u*_*0*_ the stress at the surrounding compartments, all are given by Eq. 7. *ρ* and *φ* are the radial and polar coordinates, respectively. The formation of the pore is resisted by the elongation of the highly curved pore rim. The energy per unit length needed to form the pore rim is given by *λ*, with a typical magnitude of 10–20 pN (24,75). Thus we use *λ =* 15 pN in our computations, a value that might change in specific applications depending on *J*_*sm*_ (76). The fusion pore expansion energy is the sum of rim energy minus the energy gain from lipids removal, given by

(Equation 17) $$U_{pore}\left(\rho \right)=2\pi \rho \lambda -\int_{\rho^{\prime }=0}^{\rho^{\prime }=\rho }\int_{\varphi =0}^{\varphi =2\pi }\sigma \left(\rho^{\prime },\varphi \right)\rho^{\prime }d\rho^{\prime }d\varphi \text{.}$$

To facilitate our computation, we assume that the pore formation is initiated at the center of the diaphragm and that the fast fluctuation in pore size does not change the hemifusion diaphragm shape. The fusion pore formation energy barrier is the maximum of *U*_*pore*_(*ρ*),

(Equation 18) $$E_{pore}=\max\left[U_{pore}\left(\rho \right)\right]\text{.}$$

However, since the pore must expand most of the diaphragm before it overcomes the critical energy, the initiation point is irrelevant to the *E*_*pore*_ magnitude.

#### Numerical procedures

Our numerical approach is based on previous work (77), implemented in MATLAB, and it can be downloaded at https://github.com/GonenGolani/Fusion_Solver, where further details are given. The basic idea of the computation is inspired by a finite elements approach: the fusion site is divided into membrane elements, i.e., the diaphragm, the two membranes of the initial compartments in the vicinity of the fusion site, and the two initial membranes far from the site. Each of these membrane elements is described by three vector functions: the position of the mid-plane and the upper and lower lipid directors. The components of each vector function are represented by a polynomial expansion up to order *M*, which is user-defined. The coefficients of this polynomial are set to maintain the boundary conditions. In cases where the volume is fixed, the curvature radius and fusion site sizes are determined, so the volume of the compartment is unchanged in hemifusion configurations. The energy is minimized as follows:

1) The computation starts from an initial user-defined configuration given by the set of coefficients.
2) The splay, tilt, and saddle splay are calculated based on the local geometry. In addition, the stretching energy is calculated based on the global monolayer areas.
3) The energy is a function of the polynomial coefficients and a few boundary conditions, such as the distance between fusing compartments and the size of the fusion site, which are not fixed. The global energy is minimized using a gradient descent method that operates on the coefficients. The boundary conditions on the different elements are maintained in each step.
4) The process restarts from different initial conditions to ensure the global minimum is found.

## Results

### Fusion between initially flat membranes

**Figure 4:**
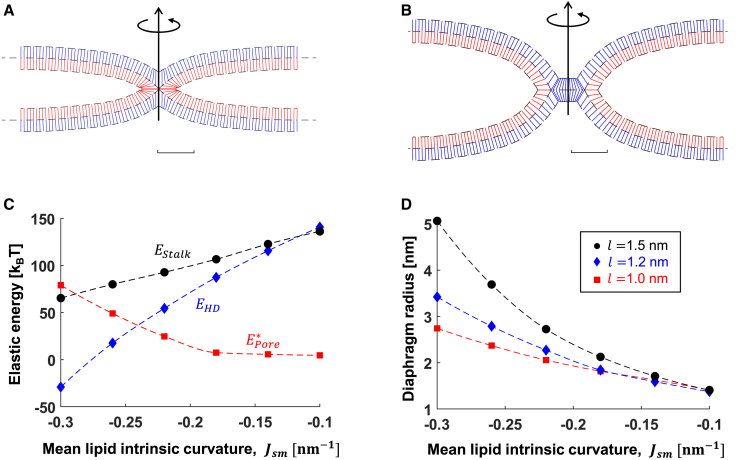
Fusion between two identical flat membrane compartments. (*A* and *B*) Simulations results of (*A*) stalk and (*B*) hemifusion diaphragm. The blue and red lines represent the averaged lipid director $\vec{n}$, blue is the distal monolayer, and red is the proximal monolayer. (*C*) Formation energies as a function of lipid mean intrinsic curvature, *J*_*sm*_: stalk (*E*_*stalk*_, *black* •), hemifusion diaphragm (*E*_*HD*_, *blue* ♦), and pore (*E*_*pore*_, *red* ▪). Tilt decay length is *l =* 1.2 nm in (*A*)–(*C*). (*D*) Equilibrium radius of the hemifusion diaphragm as the function of *J*_*sm*_ at different tilt decay lengths: black *l =* 1.5 nm, blue *l =* 1.2 nm, and red *l =* 1 nm. The parameters used in all panels are *κ*_*m*_*=* 10 k_B_T, χ = –0.5, and *δ*_*0*_ = 1.5 nm. To see this figure in color, go online.

Before considering the effect of curved geometries on the fusing pathway, we use our computational approach to simulate fusion between two flat membranes. These will be used as a reference for fusion between curved compartments in the following. We numerically simulate the shape of the hemifusion stalk (Fig. 4 *A*), hemifusion diaphragm (Fig. 4 *B*), and the energies *E*_*stalk*_, *E*_*pore*_, and *E*_*HD*_ as a function of the monolayer material parameters: *J*_*sm*_ (Fig. 4 *C*), *l* (Fig. S1 *A*), and *χ* (Fig. S1 *B*). The monolayer bending rigidity modulus, *κ*_m_, is taken as 10 k_B_T. However, its actual value ranges from 5 to 15 k_B_T (62). Thus, the energies presented in the following scale with it.

The most important factor controlling the energy barriers and the fusion rate is *J*_*sm*_ because it can be experimentally manipulated by changing the lipid composition (reviewed in (19)). For example, adding lipids with positive intrinsic curvature, such as lysophosphatidylcholine, inhibits stalk formation (78) but promotes fusion pore expansion (54), whereas lipids with negative intrinsic curvatures, such as oleic acid or diacylglycerol, promote stalk formation and inhibit pore expansion (46). In agreement with previous work (13), we found that the stalk formation energy linearly depends on *J*_*sm*_ (Fig. 4 *C*, *E*_*stalk*_), primarily due to the dependence of splay energy on *J*_*sm*_ (Fig. S1 *C*). We calculated the stalk energy range from 65 k_B_T at *J*_*sm*_ = –0.3 nm^−1^ to 136 k_B_T at *J*_*sm*_ = −0.1 nm^−1^. When comparing the hemifusion diaphragm energy (Fig. 4 *C*, *E*_*HD*_) to *E*_*stalk*_, we found that the hemifusion diaphragm configuration is favorable over the stalk only in *J*_*sm*_ > –0.11 nm^−1^ (Fig. 4 *C*, *E*_*HD*_). At lower values of *J*_*sm,*_ the stalk is a quasi-stable configuration, and hemifusion diaphragm expansion is not favorable. Therefore, in membranes with *J*_*sm*_ < −0.11 nm^−1^, such as in the case of pure phosphatidylcholine membranes (79), the fusion pore opens via a stalk-pore mechanism (47,52,80). We estimate the higher bound energy for stalk-pore opening by considering the sum of energies needed to expand the diaphragm and the energy of fusion pore opening. At *J*_*sm*_ = −0.1 nm^−1^, this energy is 9 k_B_T. However, since most biologically relevant membranes contain lipids with strong negative intrinsic curvatures, such as cholesterol and phosphatidylethanolamine, we expect that fusion reactions in most of the biological systems proceed through the expansion of the hemifusion diaphragm and the subsequent fusion pore opening. The fusion pore formation energy barrier in such cases spans from 5 k_B_T at *J*_*sm*_ = −0.11 nm^−1^ to 79 k_B_T at *J*_*sm*_ = –0.3 nm^−1^ (Fig. 4 *C*, *E*_*pore*_).

In addition to the energy barriers, the equilibrium radius of the hemifusion diaphragm, *ρ*_*D*_, is of particular interest since it can be experimentally observed. By varying *J*_*sm*_, *l* (Fig. 4 *D*)*,* and *χ* (Fig. S1 *D*, although *χ* has a small effect on *ρ*_*D*_) within the relevant range, we numerically found *ρ*_*D*_ to range from 1.4 to 5 nm. So even at the limits of the biologically relevant range (*l* = 1.5 nm and *J*_*sm*_ = −0.3 nm^−1^), the diaphragm radius is shorter than what is seen between highly curved vesicles in experiments (36,56,57). Therefore, we speculated that the curvature of the vesicles plays a significant role in determining hemifusion diaphragm radius.

To conclude, we showed that the stalk and fusion pore formation energies and hemifusion intermediate shapes between two flat membranes and their dependence on the monolayer material parameters can be calculated with continuum elastic theory. These are used as a reference for the fusion between curved compartments in the following. Moreover, we found a critical spontaneous curvature, *J*_*sm*_ = −0.11 nm^−1^, the fusion progress through the stalk-pore mechanism at higher values and diaphragm expansion at lower ones.

### Hemifusion stalk formation in curved compartments

**Figure 5:**
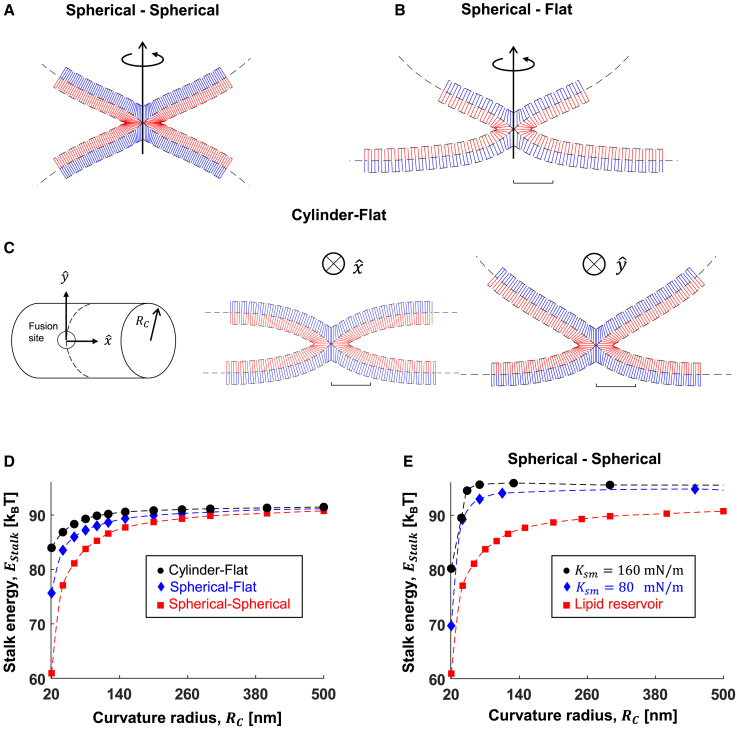
Stalk formation between curved compartments. (*A*–*C*) Simulation results of hemifusion stalk between (*A*) two identical spherical compartments, (*B*) flat and spherical compartments, and (*C*) flat and cylindrical compartments. Scale bar, 5 nm. The fusion is on the side of the cylinder in (*C*). In (*A*–*C*), the curvature radius of the compartment is *R*_*c*_. The blue and red lines represent the averaged lipid director $\vec{n}$, blue is the distal monolayer, and red is the proximal monolayer. (*D*) Stalk formation energy as a function of fusing compartment curvature radius, *R*_*c*_. No lipid stretching. (*E*) Stalk energy as a function of *R*_*c*_ in the spherical-spherical configuration. Blue, *K*_*m*_ = 80 mN/m; black, *K*_*m*_ = 160 mN/m. The red data points represent a curved membrane connected to a lipid reservoir with zero tension, so no tension-related energy is accumulated. The parameters used in all panels are *κ*_*m*_*=* 10 k_B_T, χ = –0.5, *δ*_*0*_ = 1.5 nm, and *l* = 1.2 nm. (*A*–*D*) *K*_*m*_ = 80 mN/m. To see this figure in color, go online.

We next decreased the curvature radius *R*_*C*_ in small steps from flat to 20 nm and calculated the stalk formation energy (Fig 5). We considered fusion between two vesicles (Fig. 5 *A*, spherical-spherical), a flat membrane and a vesicle (Fig. 5 *B*, spherical-flat), and between flat and cylindrical membranes (Fig. 5 *C*, cylindrical-flat). First, we considered the simplified situation with no tension, which corresponds to the fusion between two curved membranes connected to a lipid reservoir with vanishing tension that buffers the change in the area resulting from stalk formation. In agreement with previous studies (47,48,49,50), we found that the stalk energy was reduced by up to 8 k_B_T, 16 k_B_T, and 31 k_B_T for cylindrical-flat, spherical-flat, and spherical-spherical, respectively, compared with flat-flat membrane fusion (Fig. 5 *D*). In accordance with that, we found that the primary reduction in energy originates from the relaxation of splay energy, whereas the tilt and saddle splay energy contributions are almost independent of *R*_*C*_ (Fig. S2 *A*). To further assess the role of splay relaxation in stalk formation, we repeated the above procedure with different *J*_*sm*_ values (Fig. S2 *E*) and found that an additional 4.5 k_B_T reduction in *E*_*stalk*_ at *J*_*sm*_ *= –*0.30 nm^−1^ compared to *J*_*sm*_ *= –*0.10 nm^−1^ at the largest curvature simulated (flat-spherical, other configurations show similar behavior). Therefore, the combined effect of membrane curvature and intrinsic lipid curvature is minor compared with the individual effect of *R*_*c*_ (Fig 5. *D*) and *J*_*sm*_ (Fig. 4 *C*) alone. To compare with recent work by Smirnova and Müller (16), who investigated fusion with even smaller vesicles and found a strong role for hydration forces, we extended our computations in the spherical-spherical configuration down to 10 nm size vesicles with spontaneous curvature of DOPC (−0.1 nm^−1^). We found that at such high curvature, the energy of stalk formation vanishes (Fig. S2 *C* and *D*), suggesting that indeed hydration forces start to dominate.

Next, we simulated the fusion between closed membrane compartments with a fixed number of lipids and volume. The geometry of the fusing membranes plays a critical role since stalk formation results in the extension of the lipid monolayer areas and an up to 10–12 μN/m increase in tension (Fig. S2 *B*). As a result, the reduction in *E*_*stalk*_ is more moderate, between 15 and 25 k_B_T instead of 31 k_B_T (Fig. 5 *E*), in spherical-spherical configuration (and even less in spherical-flat), depending on the stretching modulus, *K*_*m*_. On the other hand, stalk formation in the cylindrical-flat configuration does not involve tension increase since all monolayers are connected to large lipid reservoirs that buffer the tension levels.

To conclude, we found that the stalk energy, *E*_*stalk*_, monotonically decreases with membrane curvature, in agreement with previous theoretical works (47,48,49). A global constraint of a fixed number of lipids and compartment volume moderates the effect but does not change the qualitative behavior.

### Expansion of hemifusion diaphragm and lipid flip-flop

**Figure 6:**
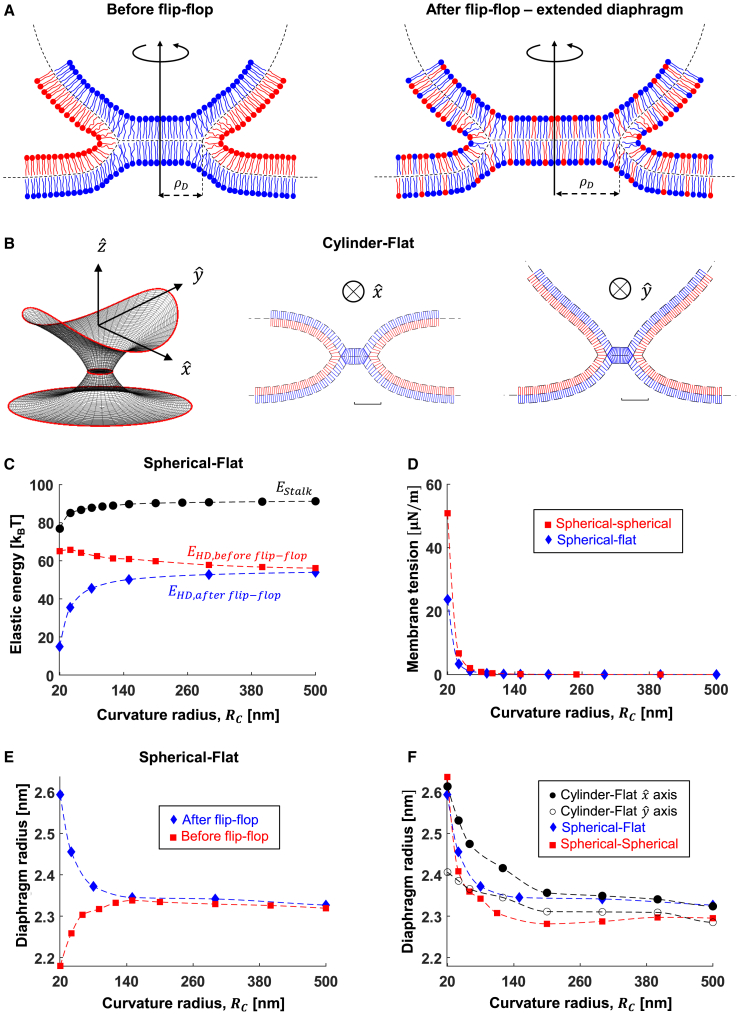
Hemifusion diaphragm geometry. (*A*) Cartoon of the hemifusion diaphragm before (*left*) and after (*right*) lipid flip-flop. The diaphragm radius in the after-flip-flop is extended compared with the before-flip-flop state. (*B*) Simulation results of the equilibrium hemifusion diaphragm formed between initially cylindrical to flat membranes. Left: position of membrane mid-planes at the fusion site. The red lines at the edges represent the area of connections to the surrounding membranes. Center: the cross-section of the hemifusion diaphragm perpendicular to $\hat{x}$, parallel to the cylinder axis. Right: the cross-section perpendicular to $\hat{y}$ axis. (*C*) Elastic energy accumulated in the stalk (*black* •) and hemifusion diaphragm, *E*_*HD*_, before lipids flip-flop (*red* ▪) and after lipids flip-flop (*blue* ♦). (*D*) Membrane tension before lipid flip-flop due to lateral lipid stretching in the spherical-spherical (*red* ▪) and spherical-flat configurations (*blue* ♦). (*E*) Diaphragm radius in the spherical-flat configuration before (*red* ▪) and after (*blue* ♦) lipid flip-flop. (*F*) Diaphragm radius in different fusing compartments geometries: spherical-flat (*blue* ♦), spherical-spherical (*red* ▪), and cylindrical-flat (*black*); solid dots (•) represent the semiminor axis in the $\hat{x}$ direction and open dots (○) in the $\hat{y}$ direction. Parameters used in all panels: *κ*_*m*_*=* 10 k_B_T, χ = **–**0.5, *δ*_*0*_ = 1.5 nm, *l* = 1.2 nm, and *K*_*m*_ = 80 mN/m. To see this figure in color, go online.

The expansion of the hemifusion stalk to a hemifusion diaphragm is driven by the relaxation of tilt and splay deformations. In curved compartments, the splay energy relaxation is more significant since the formation of a flat diaphragm is more favorable (compared with flat membranes) as the surrounding membranes are initially stressed. Stretching of the distal monolayers resists this expansion in small vesicles. The additional stretching stress can be relaxed over time due to lipid flip-flop from the proximal monolayer (81), which drives further diaphragm expansion to its final equilibrium radius (Fig. 6 *A*). Therefore, in the following, we distinguish between two temporally separated energy relaxation processes that occur after stalk formation in the fusion of vesicles. First, we consider the immediate expansion of the hemifusion diaphragm due to tilt and splay energy relaxation, accompanied by accumulation of additional stretching energy (termed “before flip-flop”). Second, we consider a slow relaxation of the stretching energy due to lipid flip-flop (termed “after flip-flop”). The speed of the first step is governed by lipid lateral flow and lateral diffusion (82), typically in the order of microseconds for a diaphragm radius of a few nanometers, whereas the second step is governed by lipid flip-flop rate with a typical time scale of seconds (83). In long tubular membranes (Fig. 6 *B*, cylindrical-flat configuration), tension does not accumulate, and the diaphragm expands in a single step.

To quantify the above process, we calculated the elastic energies after the two relaxation steps in spherical-flat (Fig. 6 *C*) and spherical-spherical (Fig. S3 *C*) configurations (examples of equilibrium shapes in Fig. S3 *A* and *B*). The energy difference between stalk to “after flip-flop” hemifusion diaphragm configurations increases with compartment curvature, making the hemifusion diaphragm more energetically stable in high curvatures. On the other hand, the “before flip-flop” configuration is becoming less stable in small *R*_*c*_ due to the increased tension (Fig. 6 *D*) and stretching energy (Fig. S3 *D*). In the more biologically common spherical-flat configuration, the stalk is expected to expand to a diaphragm of ∼2 nm radius immediately after its formation and up to an additional 0.5 nm after lipid flip-flop (Fig. 6 *E*). In contrast, the “before flip-flop” state in the spherical-spherical configuration is not favorable at R_c_ < 60 nm, and the expansion to the hemifusion diaphragm is hindered until sufficient lipid flip-flop occurs (Fig. S3 *C*).

As discussed above, cylindrical-flat fusion involves a single relaxation step between the stalk to the hemifusion diaphragm, and the diaphragm assumes an elongated elliptical shape because of its nonaxially symmetric configuration. We quantified it using the lengths of the semimajor and minor axis (along the $\hat{x}$ and $\hat{y}$ directions, respectively, Fig. 6 *B*), which we found to diverge from each other by up to 0.2 nm (Fig. 6 *F*). Therefore, the hemifusion diaphragm is still effectively circular, even in highly curved tubular membranes. Comparing the hemifusion diaphragm size at the fully relaxed state showed that the final diaphragm radius is independent of the fusing compartments geometry and reaches up to 2.6 nm in the middle of the parameter range (*l* = 1.2 nm and *J*_*sm*_ *= –*0.22 nm^−1^, Fig. 6 *F*). At longer tilt decay lengths and more negative values of *J*_*sm*_ (*l* = 1.5 nm and *J*_*sm*_ *= –*0.26 nm^−1^), our simulation results in a diaphragm radius of 6 nm (Fig. S3 *E*), which resembles the ones seen experimentally between two highly curved vesicles (36,56,57).

To conclude, the hemifusion diaphragm shape in fusion events involving small vesicles is determined by two relaxation processes: first immediate expansion due to tilt-splay stress relaxation and a slow second relaxation resulting from lipid flip-flop. The diaphragm expands by 2–6 nm in the first step and by an additional ∼0.5–1 nm in the second, depending on the *J*_*sm*_, *R*_*c*_, and *l*. As a result, tension reduces by up to 50 μN/m in the spherical-spherical configuration and by 23 μN/m in the spherical-flat configuration. In larger vesicles, this effect is much smaller. The hemifusion diaphragm size in the fully relaxed configurations resembles experiments.

### Fusion pore formation

Membrane stress in the diaphragm is the driving force for fusion pore formation. It is determined by the hemifusion diaphragm geometry (54), primarily its radius *ρ*_*D*_, and the membrane tension (84): high tension and small radius favor pore formation and vice-versa. In the previous section, we found that the tension in the distal monolayers is maximal immediately after the stalk expansion and decreases over time while the diaphragm radius increases. Therefore, we expected that the fusion pore formation energy barrier, *E*_*pore*_, would be minimal immediately after stalk expansion to the hemifusion diaphragm and increase over time.

**Figure 7:**
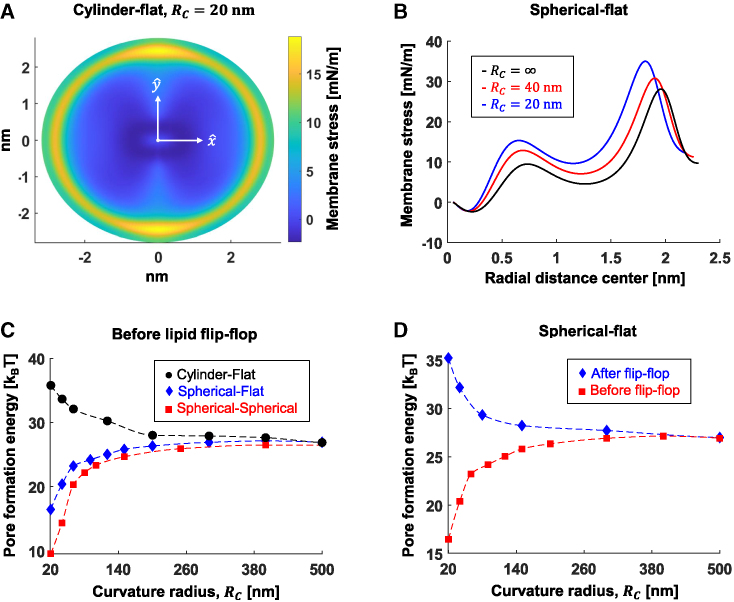
The energy barrier to fusion pore formation. (*A*) Heat map representing the membrane stress in the diaphragm formed between flat and cylindrical compartments. Cylinder radius, 20 nm. The diaphragm has an elliptical shape. (*B*) Membrane stress as a function of distance from diaphragm center in the spherical-flat configuration. Black •, infinitely large vesicles (*R*_*c*_ = ∞, flat-flat); red ▪, *R*_*c*_ = 40 nm; blue ♦, *R*_*c*_ = 20 nm. (*C*) Fusion pore formation energy barrier before lipid flip-flop at different membrane geometries. (*D*) The fusion pore formation energy barrier before (*red* ▪) and after (*blue* ♦) lipid flip-flop. Parameters used in all panels: *κ*_*m*_*=* 10 k_B_T, χ = –0.5, *J*_*sm*_ = −0.22 nm^−1^, *δ*_*0*_ = 1.5 nm, *l* = 1.2 nm, and *K*_*m*_ = 80 mN/m. To see this figure in color, go online.

We used the hemifusion diaphragm shapes found in the previous section to calculate the stresses distribution in the diaphragm (Fig. 7 *A* and *B*) as a function of the *R*_*c*_ immediately after the initial expansion of the hemifusion diaphragm (“before flip-flop”). The local stress is maximal at ∼0.5 nm from the diaphragm rim and can reach up to 20–35 mN/m, with larger stress in smaller *R*_*c*_ (Fig. 7 *A* and *B*). Thus, the fusion pore is expected to initiate near the rims, as seen in molecular dynamics simulations (18,20). Based on these stress profiles, we calculated the energy profile for fusion pore opening (Fig. S4 *A*) and *E*_*pore*_ in the different geometries and as a function of *R*_*c*_ (Fig. 7 *C*). The fusing compartment geometry plays a crucial role since, as discussed in the previous sections, the increase in tension (Fig. 6 *D*) drives pore formation in the spherical-spherical and spherical-flat but not in the cylindrical-flat configuration. As a result, *E*_*pore*_ increases by up to 9 k_B_T in the cylindrical-flat configuration compared with flat-flat, whereas it decreases by 10 k_B_T and 17 k_B_T in the spherical-flat and spherical-spherical configurations, respectively (Fig. 7 *C*). Next, we calculated *E*_*pore*_ after lipid flip-flop and found an increase of up to 19 k_B_T nm in the spherical-flat configuration (Fig. 7 *D*) and by 30 k_B_T in the spherical-spherical configuration (Fig. S4 *B*). The effect is further intensified in membranes with more negative *J*_*sm*_ values (Fig. S4 *C* and *D*) with up to an increase of 12 k_B_T at *J*_*sm*_ = −0.3 nm^−1^ in the spherical-flat configuration. On the contrary, in membranes with more positive values of *J*_*sm*_*,* the effect of curvature is negligible since the stress in the diaphragm is high and less affected by the changes in curvature.

To conclude, fusion pore formation is faster in events involving highly curved vesicles but not for tubular membranes. However, if the fusion pore does not form before the lipid flip-flop occurs, the stress relaxes in the hemifusion diaphragm, and *E*_*pore*_ increases. This process directs the fusion reaction of vesicles to a dead-end, namely an extended hemifusion diaphragm state that will be stable for a long time.

## Discussion

Here we used continuum elastic theory to calculate stalk and fusion pore formation energies, which correspond to the two main barriers determining the rate of membrane fusion. Our universal approach allows us to explore all relevant geometries on the same footing. In particular, we considered some geometries that have not been addressed before, such as the fusion of highly curved tubular membranes. We systematically varied the system parameters to evaluate how they change the pathway and included global constraints such as a constant number of lipids and volume.

We found that the stalk energy decreases with membrane curvature regardless of the fusing membrane geometry (spherical or tubular) and lipid composition. Therefore, the onset of lipid mixing after the fusion trigger is faster in highly curved vesicles, as seen in experiments (51). Our theoretical analysis also predicts that a similar effect should be seen in highly curved tubular membranes. However, to our knowledge, corresponding mixing experiments have not been performed yet.

We also predict that the hemifusion diaphragm expands in a two-step process in fusion events involving highly curved vesicles. First, a rapid expansion is driven by the relaxation of tilt and splay stresses, and second, subsequent slow relaxation occurs due to the relaxation of the stretching energy by lipid flip-flop. On the other hand, in fusion events involving long but highly curved tubular membranes, such as filopodia and microvilli, the diaphragm expands in a single fast step since it does not involve tension increase. The final diaphragm radius is larger by up to 1 nm in fusion with curvature, compared with fusion between flat membranes, and the diaphragm is less stressed. As a result, the fusion pore formation energy barrier is initially lower, and the fusion pore forms faster in vesicle fusion than in a flat membrane compartment. However, if the pore fails to open before the tension relaxes, a process that can take several seconds, the energy barrier significantly increases, and the pathway is directed to an extended hemifusion diaphragm state. At this state, the system is kinetically trapped in hemifusion. This finding explains seemingly contradicting experimental evidence: bulk fusion assay shows more efficient content mixing in highly curved vesicles (51,55). However, some remaining vesicles form extended hemifusion diaphragm structures (36,56,57), which are highly stable even at long time scales. Large curvature in tubular membranes disfavors fusion pore formation as the stress in the diaphragm is lower compared with the fusion of flat membranes. Therefore, the content mixing rate is slower in a highly curved membrane connected to a lipid reservoir that can buffer the change in tension.

**Figure 8:**
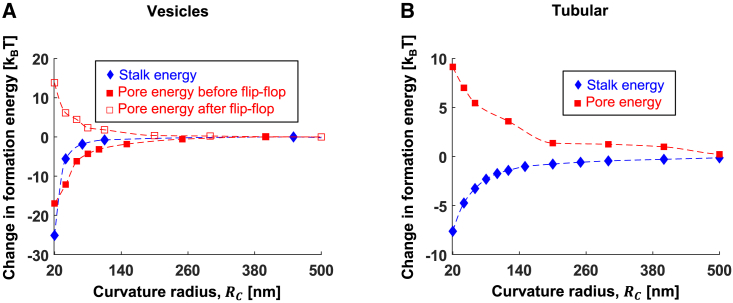
The dependence of stalk and fusion pore formation energy barriers on the compartment curvature radius. (*A*) Vesicle-vesicle fusion. (*B*) Fusion of flat and initially cylindrical membrane. Parameters: *κ*_*m*_*=* 10 k_B_T, χ = –0.5, *J*_*sm*_ = −0.22 nm^−1^, *δ*_*0*_ = 1.5 nm, *l* = 1.2 nm, and *K*_*m*_ = 80 mN/m. To see this figure in color, go online.

We next discuss the biological relevance of our results. The time needed to complete the fusion reaction in biological systems varies across scales, from microseconds to minutes, depending on the mechanical work of the specific fusion proteins involved, their abundance, and the local membrane mechanical properties. Moreover, the time needed to cross the two energy barriers of lipid and content mixing varies significantly between different systems and is not well known since it is experimentally challenging to follow both the lipids and the content of the fusing compartments. These two typical times are particularly important in the context of membrane geometry since stalk and pore formation energy barriers are affected differently by the membrane geometry (Fig. 8): stalk formation is always more favored in large curvature. In contrast, pore formation is only favored in fast fusion events involving vesicles, such as SNARE-mediated synaptic fusion, but not in slow fusion events, such as influenza A hemagglutinin-mediated fusion, or events involving fusion with long tubular membranes.

An example of a fast fusion event is synaptic vesicle fusion, which typically takes microseconds to complete (85). In accordance with our results, the logarithm of fusion pore formation time was found to increase linearly with the curvature radius of small vesicles in vitro (46,86). This indicates that the fusion pore formation rate follows a simple Arrhenius law with the mean-time of pore opening given by *τ*_*pore*_ (87):

(Equation 19) $$\tau_{pore}=C\cdot exp\frac{E_{pore}}{k_{B}T}\text{.}$$

Although our computations do not allow us to predict the time of the fusion event, since we do not know the preexponential factor *C*, we can estimate the change in mean formation time as a function of vesicle size using our numerical results. For example, the fusion pore formation energy barrier for a 20-nm-radius vesicle is lower by ∼5 k_B_T than for a 40-nm-radius vesicle, resulting in a 150 times faster fusion pore formation rate. So smaller vesicles complete the fusing reaction much faster, as observed experimentally (46,86).

Another important example of the role of fusing compartment geometry is in enveloped viruses, which must fuse their lipid envelope with that of the cell to infect it. We first note that to prevent infection, the host could direct the fusion of the viral membrane into the dead-end predicted here that occurs after lipid flip-flop. Second, we note that some enveloped viruses, such as influenza A and Ebola, are polymorphic; they can be either spherical or tubular. The curvature radius of the tubular influenza A virus was measured to be 20 nm, and spherical ones are ∼40 nm (88). Recent experimental evidence shows that the infectivity of tubular virions is higher than spherical ones (43). However, based on our numerical computations, we conclude that this cannot be explained by the change in energy barriers, as the stalk energy is almost unchanged between 20 nm tubular to 40 nm spherical particles (*E*_*stalk*_ ≈ 83 k_B_T, Fig. 5 D) and the fusion pore formation energy barrier decreases by ∼3 k_B_T in spherical virions. We speculate that the main reason for this observation lies elsewhere, e.g., that tubular viruses explore space differently and might have more opportunities to fuse due to higher surface area and higher numbers of viral fusion proteins per single virion. Alternatively, a theoretical analysis predicted a higher uptake rate of tubular particles over spherical ones (89), suggesting that endocytosis might be the important step affected by virus shape. Moreover, spherical and tubular virions form in different pathways, resulting in distinct lipid compositions and structures viral matrix layer, which can also affect the fusion rate (90). Therefore, further research is needed to clarify the relationship between virus shape and infectivity.

Lastly, local membrane curvature plays a crucial role in cell-cell fusion, for example, of egg and muscle cells, with fusion preferably occurring in highly curved membrane tubular structures such as the microvilli. Our analysis showed that fusion with these plasma membrane domains has a lower stalk formation energy but higher fusion pore formation energy. However, the fusion protein density on the plasma membranes is much lower than in synaptic vesicles and enveloped viruses. Therefore, the initial formation of the stalk is expected to be the primary barrier in the process, and the gain of reducing the stalk formation energy outweighs the loss of increasing fusion pore formation energy. The importance of microvilli curvature in oocyte-sperm fusion was experimentally illustrated by knocking out the CD9 protein, which increased the microvilli curvature radius from 38 nm to 70 nm (45) and reduced the fusion rate (91). In our model, the stalk formation energy increases by 2.2 k_B_T in the cylindrical part of the microvilli and by 3.6 k_B_T in the spherical tip due to this curvature change (Fig. 5 *D*), making the stalk formation slower by a factor of 10–36, depending on the fusion location in the microvilli. Regardless of CD9 knockouts, the fusion preferably occurs at microvilli since the energy of stalk formation is lower by 15.5 k_B_T at the tip compared with the flat parts of the plasma membrane and by 7.2 k_B_T from the cylindrical part. Therefore, cell-cell fusion is much preferable in these highly curved membrane domains.

Our modeling does not treat the effect of membrane curvature on alternative fusion pathways to the canonical one as described in the introduction and depicted in Fig. 1 (reviewed in (92)). For example, based on computer simulations, it was suggested that the hemifusion stalk is elongated rather than radially expanded, known as the “elongated stalk” pathway (14,27,28,30). Although our model allows nonaxially symmetric diaphragms, we do not allow for elongation of the stalk. However, a previous study that is based on a similar continuum elasticity approach found that this expansion is possible in very low *J*_*sm*_ conditions (<–0.4 nm^−1^) and is not favorable over radial expansion (17). Determining the curvature’s effect on this pathway’s favorability is beyond the scope of this work. In addition, the compartment curvature is also expected to induce the formation of stable membrane pores outside of the diaphragm, for example at the stalk or later at the vertex line, known as “leaky fusion” (56). The stabilization of such pores is more likely in highly curved vesicles because of the increased stress in the membranes outside the diaphragm and might also be favored by fusion proteins. These are possible explanations for the membrane pores seen in the fusion of liposomes with influenza A virus-like particles in vitro (56). However, similar pores or leaks are not seen in the fusion of spherical influenza A viruses in the endosome in vivo (93). Therefore, it is unknown whether the curvature alone can induce leaky fusion, and further theoretical research is needed.

In conclusion, the fusion of curved membrane domains is crucial for many biological systems, and our theory provides a unifying treatment for the different physical barriers to fusion. However, biological membranes differ not only in geometrical shape and lipid compositions but also in many other important aspects, such as the presence of curvature-inducing proteins, asymmetry in lipid compositions, and preexisting tension. Finally, our continuum approach does not consider hydration forces, which play an important role when membranes are forced into close opposition. These factors, which were not discussed here, determine the local membrane mechanics and will also contribute to the energy barriers for membrane fusion and fission. Depending on the system of interest, the framework presented here can now be used to disentangle the different factors at play during the fusion of curved membranes.

## Author contributions

G.G. and U.S.S. designed the research. G.G. performed the research. G.G. and U.S.S. wrote the manuscript.

## Supporting material

Document S1. Figures S1–S4

Document S2. Article plus supporting material
