# Supplementary material for: High curvature promotes fusion of lipid membranes: Predictions from continuum elastic theory
Source: Biophys J. 2023 Apr 18;122(10):1868–82. doi: 10.1016/j.bpj.2023.04.018 (PMC10209146; doi:10.1016/j.bpj.2023.04.018)
Supplement: Document S1. Figures S1–S4 [file mmc1.pdf]

Biophysical Journal, Volume *122*

Supplemental information

High curvature promotes fusion of lipid membranes: Predictions from continuum elastic theory

Gonen Golani and Ulrich S. Schwarz

Supplementary figures

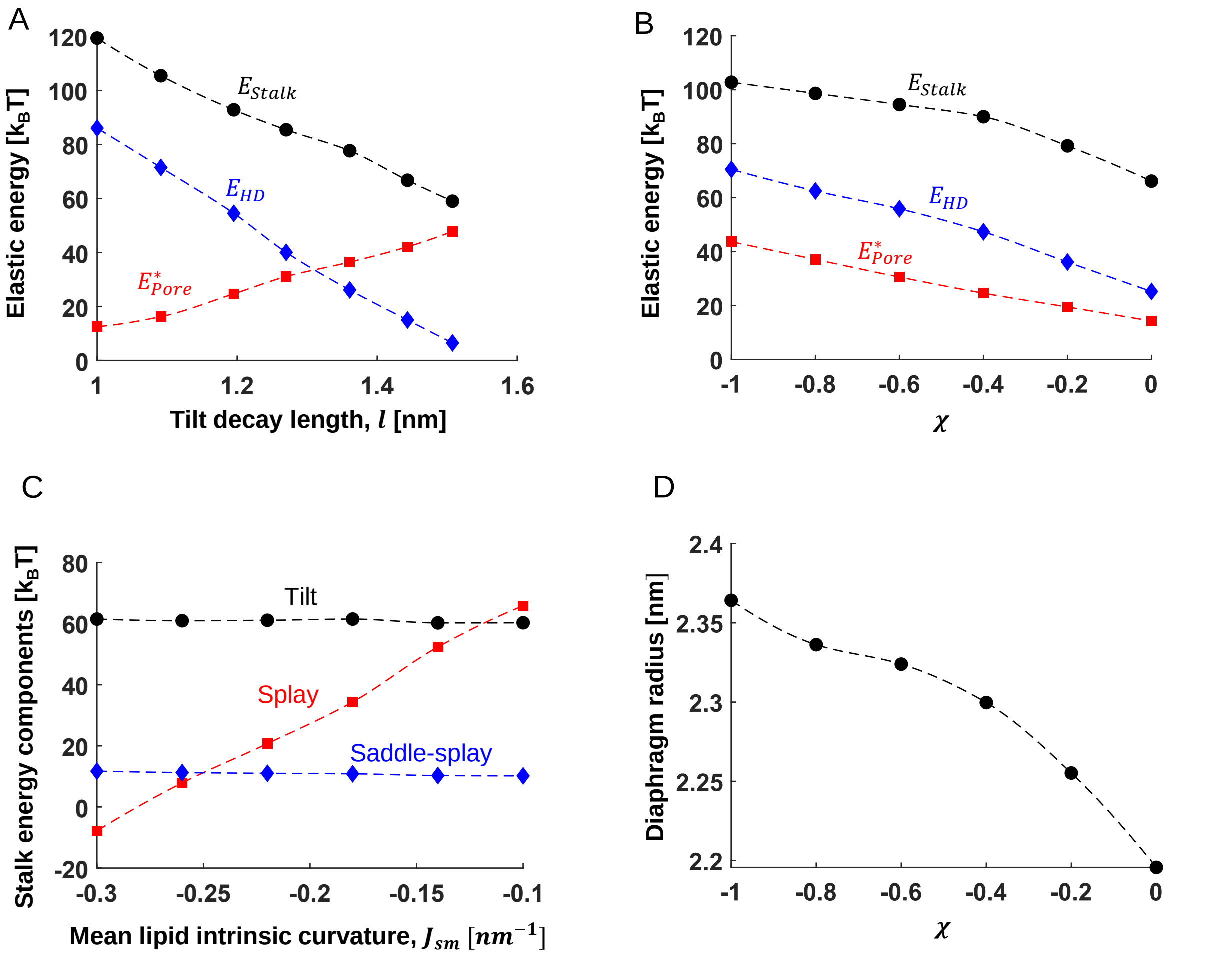

Figure 1S - **Fusion between two identical flat membrane compartments, supplement to figure 4**. (A-B) Formation energies for stalk (E_{stalk}, black ●), hemifusion diaphragm (E_{HD}, blue ♦), and pore (E_{pore}, red ■). (A) As a function of tilt decay length $l = \sqrt{\kappa_m/\kappa_t}$. Fixed parameters: χ = -0.5 and J_{sm}=-0.22 nm^{-1}. (B) As a function of the ratio between monolayer saddle-splay to bending modulus, $\chi = \bar{\kappa}_m/\kappa_m$. Fixed parameters: l=1.2 nm and J_{sm}=-0.22 nm^{-1}. (C) The energy contribution of the different lipid deformations as a function of J_{sm}: Tilt (black ●) - $\int \frac{1}{2}\kappa_t t^2 dA$, saddle-splay (blue ♦) - $\int \bar{\kappa}_m \tilde{K} dA$ and splay (red ■) - $\int \frac{1}{2}\kappa_m(\tilde{J}^2 - 2\tilde{J}J_{sm})dA$. The integration is done over all monolayers. Fixed parameters: χ = -0.5 and l=1.2 nm. (D) Diaphragm radius as a function of χ. Parameters: J_{sm}=-0.22 nm^{-1}and l=1.2 nm. In all panels: δ_0=1.5 nm and κ_m=10 k$_B$T.

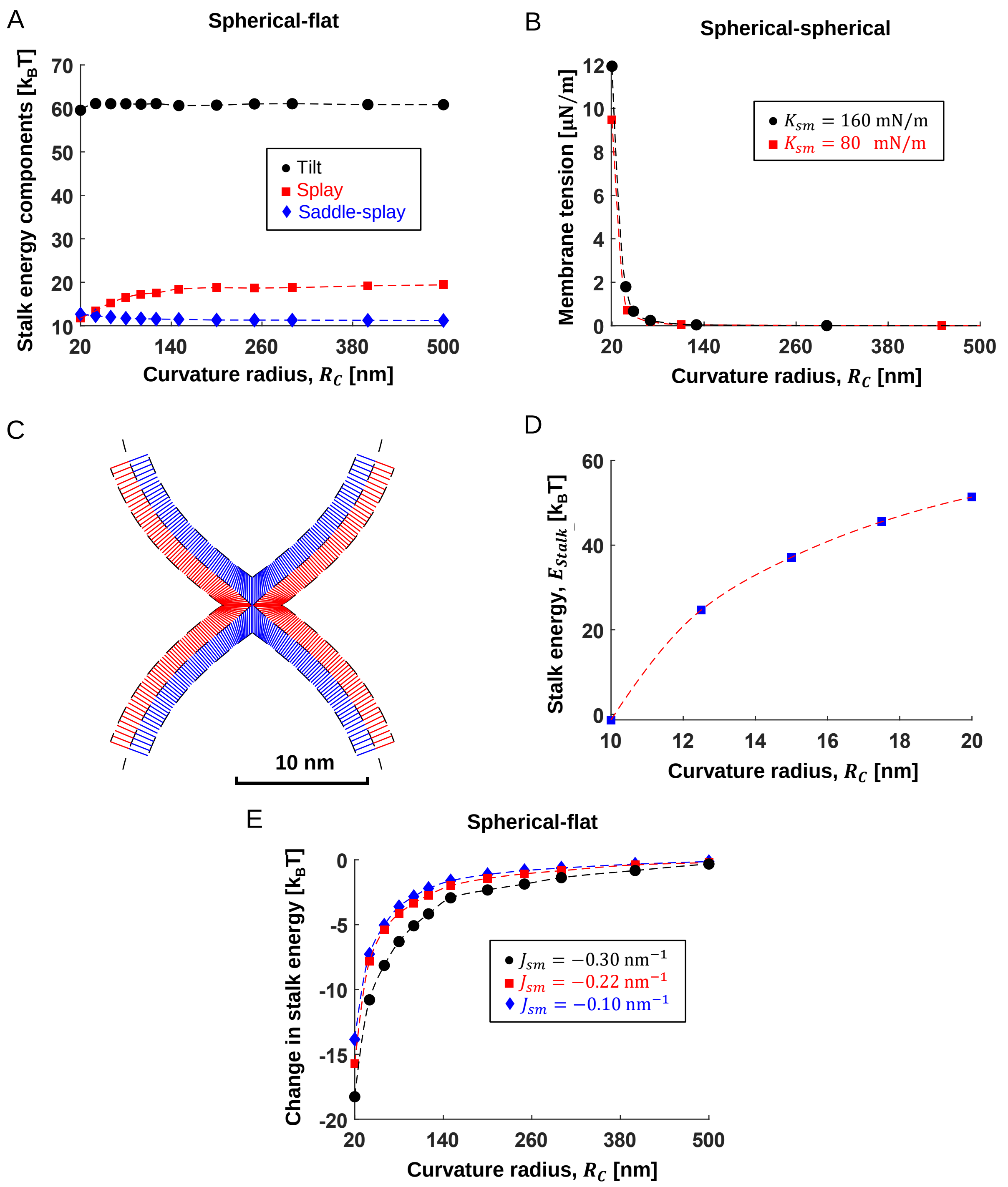

Figure 2S - **Stalk formation between curved membranes, supplement to figure 5**. (A) The energy contribution of the different lipid deformations as a function of R_c: Tilt (black ●) - $\int \frac{1}{2}\kappa_t t^2 dA$, saddle-splay (blue ♦) - $\int \bar{\kappa}_m \tilde{K} dA$ and splay (red ■) - $\int \frac{1}{2}\kappa_m(\tilde{J}^2 - 2\tilde{J}J_{sm})dA$. The integration is done over all monolayers. No tension, spherical-flat configuration. (B) Membrane tension as a function of R_c with different lipid monolayer stretching modulus, K_m, spherical-spherical configuration. The parameters used in (A) and (B) κ_m=10 k_BT, χ=-0.5, δ_0=1.5 nm, and l=1.2 nm. (A-B) K_m=80 mN/m. (C and D) Highly curved pure DOPC spherical-spherical fusion. Parameters: κ_m=10 k_BT, χ=**0**, δ_0=1.5 nm, and l=1.5 nm. (C) The Hemifusion stalk shape at R_c=10 nm, (D) stalk energy as a function of the vesicles curvatures radius. (E) Change in stalk energy compared as a function of

R_c for different values of J_{sm}, spherical-flat configuration. Reference stalk energies (Rc = ∞): 65 k_BT, 92 k_BT, and 123 k_BT for J_{sm}=-0.30 nm^{-1} (black ●), J_{sm}=-0.22 nm^{-1} (red ■), and J_{sm}=-0.1 nm^{-1} (blue ♦), respectively.

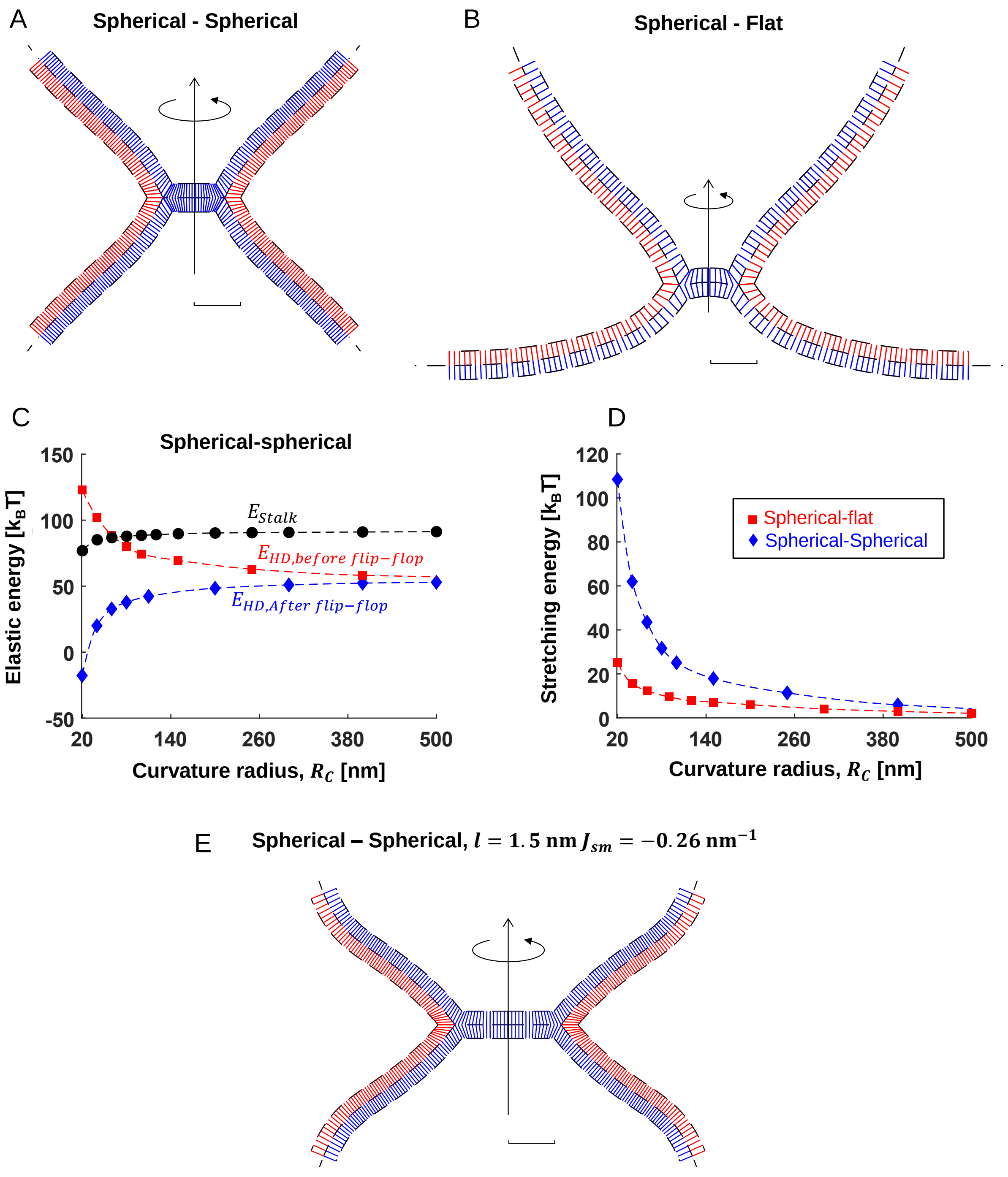

Figure S3 - **Hemifusion diaphragm geometry, supplement to figure 6.** (A-B) Simulation results of equilibrium hemifusion diaphragms. The blue and red lines represent the averaged lipid director $\vec{n}$, blue is the distal monolayer, and red is the proximal monolayer. (A) Spherical-spherical configuration (B) Spherical-flat configuration. Parameters: fixed volume, after lipid flip-flop and R_c=20 nm. (C) Elastic energy accumulated in the stalk (E_{stalk}, black ●) and hemifusion diaphragm, E_{HD}, before lipid flip-flop (red ■) and after lipid flip-flop (blue ♦) at the spherical-spherical configuration. (D) Stretching energy, $\int \gamma dA$, as a function of R_c in the

spherical-flat (red ■) and spherical-spherical (blue ♦) configurations. (E) Hemifusion diaphragm equilibrium shape after flip-flop, an example of extended hemifusion diaphragm. Parameters in (A-D) κ_m=10 k_BT, χ=-0.5, δ_0=1.5 nm, l=1.2 nm, J_{sm}=-0.22 nm^{-1} and K_m=80 mN/m. Only in (E): l=1.5 nm and J_{sm}=-0.26 nm^{-1}.

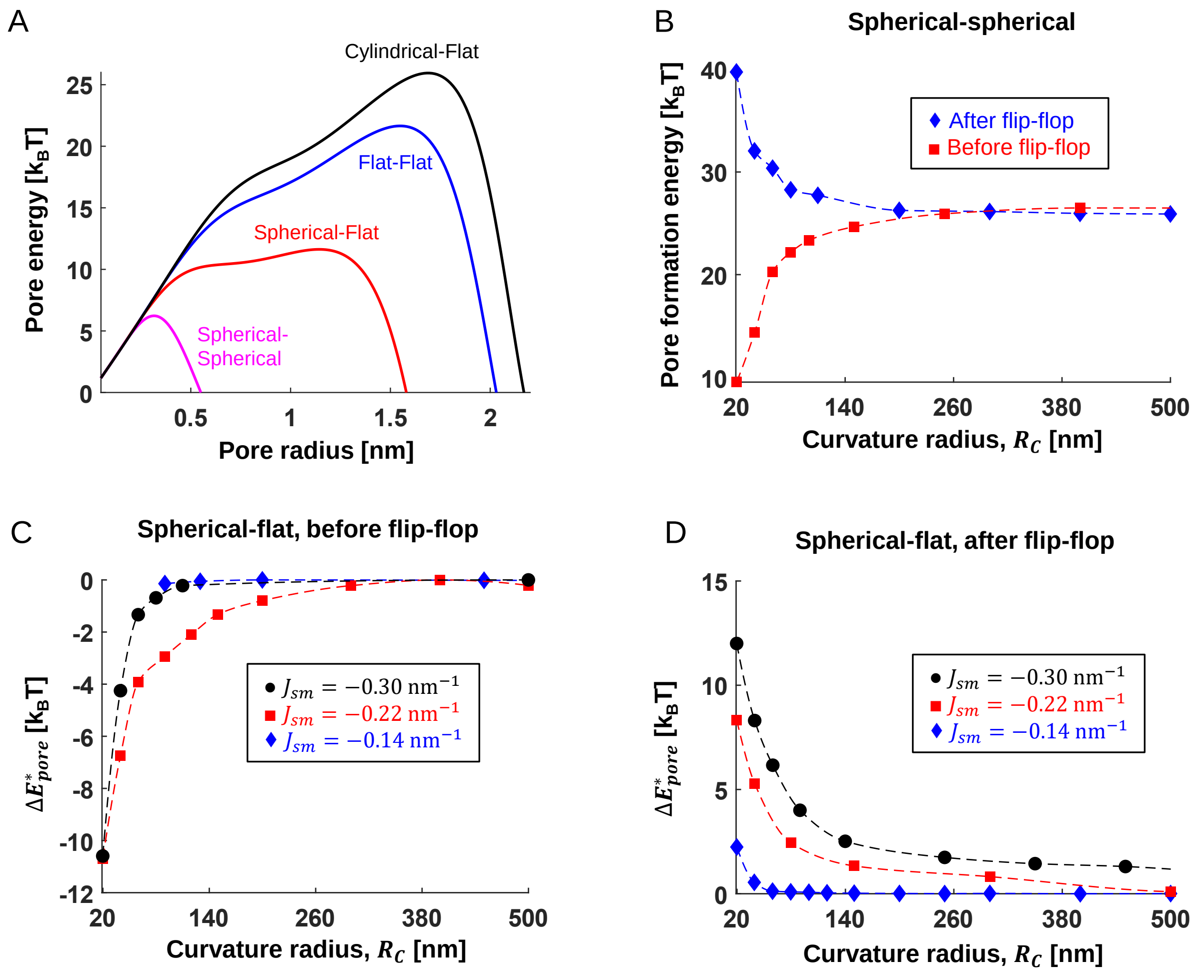

Figure S4 - **Fusion-pore formation energy barrier, supplement to figure 7.** (A) Pore energy as a function of pore radius in the different configurations. (B) Pore formation energy barrier, E_{pore}, as a function of R_c before (red ■) and after (blue ♦) lipid flip-flop. (C-D) Change in E_{pore} compared to the flat-flat configuration as a function of R_c for different values of J_{sm}. Reference values of E_{pore} are 79 k_BT, 25 k_BT, and 5.6 k_BT for J_{sm}=-0.30 nm^{-1} (black ●), J_{sm}=-0.22 nm^{-1} (red ■), and J_{sm}=-0.14 nm^{-1} (blue ♦), respectively. (C) Before lipid flip-flop, (D) after flip-flop. Parameters in all panels: κ_m=10 k_BT, χ=-0.5, δ_0=1.5 nm, l=1.2 nm, and K_m=80 mN/m. Only in (B) J_{sm}=-0.22 nm^{-1}.
